# Cell senescence, the senescence-associated secretory phenotype, and cancers

**DOI:** 10.1371/journal.pbio.3002326

**Published:** 2023-09-21

**Authors:** Larissa G. P. Langhi Prata, Tamar Tchkonia, James L. Kirkland

**Affiliations:** 1 Department of Physiology and Biomedical Engineering, Mayo Clinic, Rochester, Minnesota, United States of America; 2 Department of Medicine, Mayo Clinic, Rochester, Minnesota, United States of America

## Abstract

In this Perspective, the authors look back at a 2008 PLOS Biology article that reported a senescence-associated secretory phenotype that could promote inflammation and cancer, and discuss how that discovery eventually enabled the development of senolytic drugs.

## Teaser

Cellular senescence is a cell fate caused by multiple stresses. A 2008 article in *PLOS Biology* reported a senescence-associated secretory phenotype that can promote inflammation and cancer, eventually enabling the development of senolytic drugs.

This article is part of the *PLOS Biology* 20th Anniversary Collection.

Cellular senescence was first described in 1961 by Hayflick and Moorehead [1]. At first, it was dismissed by many as a phenomenon or artefact related to repeated replication of cultured cells. The relevance of cellular senescence to human health took decades to uncover. It is now appreciated that senescent cells, which exist in a state of essentially irreversible replicative arrest, can occur in response to stresses such as telomeric dysfunction, DNA damage, oncogene expression, metabolic stresses, cytotoxic chemotherapy, radiation, and many others [2]. Almost any vertebrate cell type, including terminally differentiated cells and cancer cells, can become senescent. Senescence can be beneficial: it appears important in tissue remodeling, such as after injury or during embryogenesis and, importantly, can serve as a defense against cancer development. Indeed, preventing the generation of senescent cells by interfering with expression or activity of senescence-inducing regulators (such as p16^INK4a^, Rb, p21^CIP1^, and p53) can accelerate cancer development. However, persisting senescent cells can be harmful and appear to be root cause contributors to multiple disorders and diseases across the lifespan that account for considerable morbidity and the bulk of health care expenditures [3]. Beneficial effects of being able to form senescent cells when needed may have evolved at the expense of adverse effects caused by persisting senescent cells in older, post-reproductive individuals, who have lived beyond the point at which natural selection principally acts [4,5].

Senescent cells are metabolically active, quite resistant to cell death and, as reported in a seminal 2008 paper in *PLOS Biology* by Coppé and colleagues [5], express a senescence-associated secretory phenotype (SASP). As shown in that paper, the SASP takes 4 days or more to develop fully in cultured fibroblasts. It can include a range of factors that leads to damage, inflammation, fibrosis, metabolic dysfunction, spread of senescence to other cells, and multiple other effects at the tissue level. Coppé and colleagues showed overlapping elements of the SASP across different cell types that had been induced to become senescent, as well as among inducers of senescence (e.g., repeated replication versus radiation). Furthermore, they showed that the SASP can occur in vivo. Mitoxantrone is a DNA-damaging chemotherapy drug for prostate cancer that can induce cellular senescence: Coppé and colleagues found SASP factor release to be higher in cells isolated from patients after receiving mitoxantrone compared to before such treatment, indicating the SASP occurs in people.

Senescent cells are relatively resistant to cell death and are normally removed by the immune system. Coppé and colleagues found, among other SASP factors, chemokines and other proteins that can attract, activate, and/or anchor immune cells. If the immune system is dysfunctional, senescent cells can persist. Coppé and colleagues speculated, and now many groups have confirmed, that the SASP of persisting senescent cells contributor to multiple acute and chronic disorders and diseases, the geriatric syndromes (including frailty, cognitive dysfunction, and immobility), and decreased physical resilience (e.g., impaired recovery after infection or trauma) [6]. Coppé and colleagues [5] showed elegantly that the SASP of these persisting senescent cells can promote cancer progression, with epithelial to mesenchyme transitions (EMTs) and increased invasiveness in a paracrine manner that is dependent, in part, on the SASP factors IL-6 and IL-8. Hence, entry of damaged and potentially cancerous mutation-harboring cells into the senescent cell fate can serve as a defense against cancers due to replicative arrest, inflammation, and attraction of immune cells. These immune cells can kill not only the SASP-expressing, cancer-harboring senescent cells, but potentially also other nearby cancerous cells. However, persisting senescent cells appear to promote cancer progression and invasiveness, in part through the inflammatory microenvironment they create, but also by inducing fibrosis and causing impaired circulation that can shield cancers from immune cells. Coppé and colleagues went on to show that the pro-malignant features of the SASP of persistent senescent cells are exacerbated in cells that express high levels of the oncogene RAS and in cells with reduced function of p53. Decreasing p53, which can occur during cancer progression, can therefore exacerbate the pro-carcinogenic SASP and further promote cancer progression, potentially fueling a vicious cycle. These findings have helped to drive the current call for studies into the potential benefits of cycling chemotherapy or radiation with agents that target senescent cells and/or the SASP [7,8]. These agents include drugs that selectively remove senescent cells (known as senolytics) and drugs that interfere with the SASP (known as senomorphics), such as metformin or rapamycin.

The discovery of senolytics [9] was enabled by 3 observations: the finding that senescent cells are relatively resistant to programmed cell death (apoptosis) by Eugenia Wang in 1995 [10]; studies by Norman Sharpless and colleagues in 2004 [11] that associated increases in healthspan with reduced age-related senescent cell accumulation in calorically restricted or growth hormone receptor deficient mice; and the discovery of the SASP by Coppé and colleagues in 2008 [5]. Efforts to develop senolytics began in 2004 shortly after publication of the Krishnamurthy and colleagues article [11] with attempts to create fusion proteins between toxins and antibodies that would theoretically bind to senescent cell-specific antigens (such as that described by James Smith and colleagues in 1990 [12]) and kill senescent cells. However, neither that approach nor multiwell cell culture screens for compounds that would selectively kill senescent cells were successful initially. The Coppé and colleagues article [5] about the SASP that can include pro-apoptotic factors and the Wang article [10] showing senescent cells survive, despite damaging the cells around them, led to the question of whether transiently disabling those senescent cells with a pro-apoptotic SASP would lead them to commit “suicide” while leaving non-senescent cells unharmed [9]. Using proteomic data and bioinformatics methods, several such senescent cell anti-apoptotic pathways were identified. Their importance for protecting senescent cells from their own SASP was demonstrated using RNA interference to briefly disable key parts of these protective anti-apoptotic pathways. Natural products and drugs already in human use that targeted those key parts were found to be senolytic: they selectively killed subsets of senescent cells, depending on the characteristics of their SASP. The Campisi group was closely involved in identifying additional senolytics [13,14].

**Fig 1:**
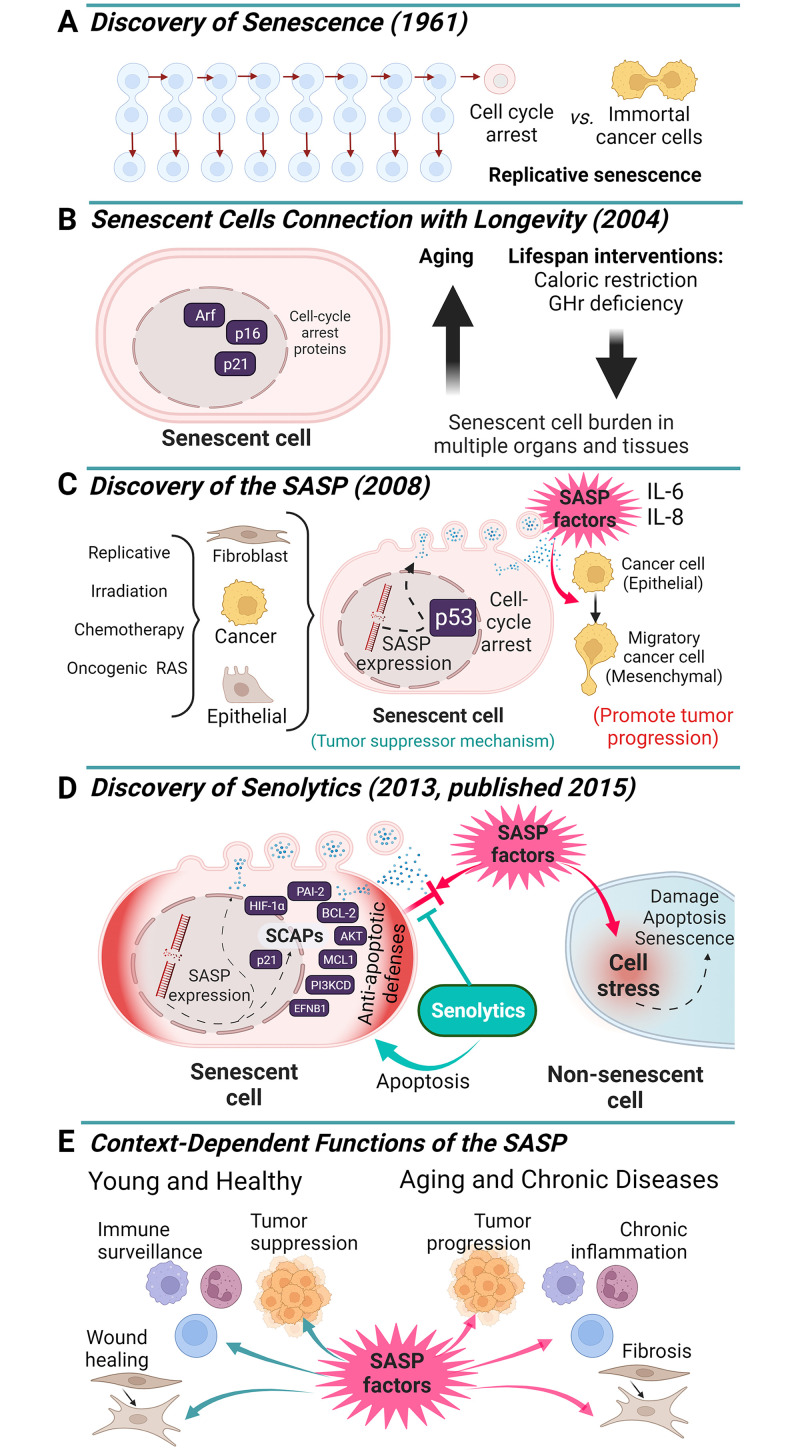
Key discoveries about cellular senescence that enabled development of senolytic interventions. The discoveries of replicative senescence in non-cancerous cells by Hayflick and Moorehead in 1961 [1] (**A**), that senescent cells are resistant to apoptosis in 1995 [6], that decreased senescent cell abundance, increased healthspan, and decreased senescent cell abundance are inter-linked in 2004 [7] (**B**), and of the SASP by Coppé and colleagues in 2008 [1] (**C**), paved the way for the discovery of senolytics [5] (**D**). Furthermore, Coppé and colleagues [1], and others, made the important observations that the SASP can cause bystander damage to non-senescent cells, modulate the immune system, cause tissue remodeling, and affect tumors context-dependently (**E**). With respect to cancers, Coppé and colleagues noted that capacity to generate senescent cells is a tumor defense mechanism. However, persisting senescent cells can promote cancer progression and invasiveness. This is an example of antagonistic pleiotropy: a beneficial process in younger individuals can be harmful at post-reproductive older ages. Based on the discovery of the SASP by Coppé and colleagues and the knowledge about it later used in devising a hypothesis-driven approach for discovering senolytic agents, there are currently over 30 early phase clinical studies of senolytics underway for treating multiple disorders and diseases. These include clinical studies of senolytics for targeting cancers and the side effects of DNA damaging cancer treatments, such as radiation and cytotoxic chemotherapy. Created with BioRender.com.

Many senolytic agents have now been identified. These drugs appear to delay, prevent, alleviate, or treat over 70 disorders and diseases in preclinical models and there are currently multiple clinical trials of senolytics underway [6]. Very early results are indicating safety, tolerability, and reduced senescent cell abundance in humans, but much more remains to be done to test the effectiveness of senolytics for alleviating senescence-associated diseases. At this point, in our view, these agents should only be used in carefully regulated clinical studies. Unless and until some of the trials are successful, senolytics will not be ready for general clinical use, but at least the field has progressed to the point of Phase 2a clinical trials. Hence, the publication in *PLOS Biology* by Coppé and colleagues was a highly important milestone (Fig 1) along the path to the discovery of senolytic agents and for beginning to translate them into potential interventions for multiple disorders that are currently difficult to treat, including many cancers.
